# Field-induced exciton condensation in LaCoO_3_

**DOI:** 10.1038/srep30510

**Published:** 2016-07-27

**Authors:** A. Sotnikov, J. Kuneš

**Affiliations:** 1 Institute of Physics, Academy of Sciences of the Czech Republic, Na Slovance 2, 182 21 Praha 8, Czech Republic

## Abstract

Motivated by recent observation of magnetic field induced transition in LaCoO_3_ we study the effect of external field in systems close to instabilities towards spin-state ordering and exciton condensation. We show that, while in both cases the transition can be induced by an external field, temperature dependencies of the critical field have opposite slopes. Based on this result we argue that the experimental observations select the exciton condensation scenario. We show that such condensation is possible due to high mobility of the intermediate spin excitations. The estimated width of the corresponding dispersion is large enough to overrule the order of atomic multiplets and to make the intermediate spin excitation propagating with a specific wave vector the lowest excitation of the system.

Perovskite cobalt oxide LaCoO_3_ exhibits unusual physical properties that have attracted attention for over half a century. Fine balance between crystal field splitting and ferromagnetic Hund’s coupling places the Co^3+^ ions to the vicinity of a spin-state transition. In combination with rather covalent Co-O bonds and electron-lattice coupling it makes LaCoO_3_ a complicated physical system. The electrical conductivity and magnetic susceptibility divide the *T*-dependent phase diagram of LaCoO_3_ into three regions with crossovers in-between: diamagnetic insulator (<80 K), paramagnetic insulator, and paramagnetic metal (>600 K). Several approaches have traditionally been used to describe the physics of LaCoO_3_: (i) the single-ion picture of low spin (*S* = 0, LS) ground state of the Co^3+^ ion with the intermediate spin (*S* = 1, IS) or high spin (*S* = 2, HS) excitations^1^,^2^ augmented with spin-exchange between these states on the lattice^3^, (ii) band structure approaches with electrons interacting via static mean field^4^,^5^,^6^ and (iii) combination of both in the form of dynamical mean-field theory^7^,^8^,^9^. Despite numerous studies, underlying physics of LaCoO_3_ remains an open problem.

The intermediate temperature regime with a paramagnetic susceptibility, a charge gap and no sizeable Co-O bond-length disproportionation is particularly difficult to describe. Existing theories either yield a metallic state^5^ or exhibit a spin-state order (SSO)^2^,^10^,^11^,^12^, a periodic arrangement of Co atoms in different spin states necessarily accompanied by Co-O bond-length disproportionation. Recent high magnetic fields experiments^13^,^14^,^15^, which found metamagnetic transition above 50 T, provide an important clue as to the nature of the intermediate temperature regime. As pointed out by authors of ref. 15 the increase of the critical field *h*_*c*_ with temperature *T* is counterintuitive given the fact that increasing temperature promotes population of the magnetic states.

In this paper we show that mobility of IS excitations on the LS background, an aspect missing in existing theories, plays an important role in LaCoO_3_. Estimates based on first principles calculations show that while the HS excitations are essentially immobile, the bandwidth of the IS dispersion is of the order of several 100 meV. The lowest excitation in solid, therefore, may have different character than the lowest single-ion excitation as depicted in Fig. 1. The nature of the low-lying excitations can be probed by the field-induced transition. Immobile excitations favour formation of SSO. Mobile excitations, on the other hand, lead to formation of a homogeneous excitonic condensate (EC). While both SSO and EC can be induced by magnetic field, we show that their *h*_*c*_(*T*) dependencies have opposite slopes.

In the following, we use dynamical mean-field theory (DMFT) to study the minimal model allowing for a spin-state transition^16^. We show that both SSO and EC can be induced by an external magnetic field and calculate the temperature dependencies of the critical field *h*_*c*_(*T*) together with other relevant physical observables close to the phase boundaries. To demonstrate the feasibility of the EC scenario, we use density functional band structure analysis to estimate the dispersion of the IS and HS excitations in real material.

## Model

We study the two-orbital Hubbard model on a square lattice, which exhibits both the instability towards formation of SSO and EC^16^. With an external magnetic field *h* the Hamiltonian reads


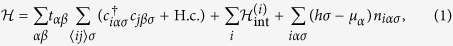


where 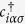 (*c*_*iασ*_) are the fermionic creation (annihilation) operators acting at the lattice site *i*, *α* = {*a*, *b*} represents the orbital index, *σ* = {1/2, −1/2} denotes the electron spin projection on the magnetic-field axis (in units of *ħ* = 1), the 2 × 2 symmetric matrix *t*_*αβ*_ consists of the amplitudes for the usual (intra-orbital, *α* = *β*) and the cross (inter-orbital, *α* ≠ *β*) hopping processes, and the notation 〈*ij*〉 indicates the summation only over nearest-neighbour sites. The local interaction part 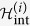 is chosen to have only density-density contributions,


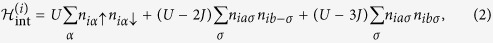


and in the last term *μ*_*a*,*b*_ = *μ* ± Δ/2, where *μ* is fixed to yield average filling of two electrons per lattice site and Δ is referred to the crystal-field splitting.

We use dynamical mean-field theory (DMFT)^17^ with the continuous-time quantum Monte Carlo hybridization-expansion (CT-HYB) impurity solver^18^,^19^ in the so-called segment representation modified to include off-diagonal hybridization important for an account of the excitonic instability close to the spin-state crossover^16^,^20^. This approach allows to obtain the Green’s functions of the impurity problem, therefore, gives an access to spectral characteristics and corresponding local observables, e.g., the correlators 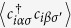 that provide with information on the magnetization (by taking the elements with *α* = *β*) and the EC order parameters (*α* ≠ *β*). In the homogeneous case (single unit cell) we focus on the EC order parameters of the type 
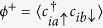
 and 
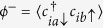
 and the magnetization 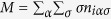 with 
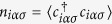
. In case of SSO, we also study the difference in occupations of the *a*-orbital on two neighbouring sites *i* and *j*, 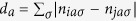.

First, we analyse the phase diagram of EC in the external magnetic field. We start from parameters of ref. 20 where the excitonic condensation was found around 850 K. We increase the crystal field so that the transition is suppressed, see Fig. 2a, and choose the crystal field Δ so that the system is in the normal [N(LS)] phase, but close to the phase boundary. Then, we apply an external magnetic field *h*. Upon reaching a critical field *h*_*c*_ the system undergoes a transition to the ferromagnetic excitonic condensate characterized by non-zero value of |*ϕ*^+^| > |*ϕ*^−^| for positive *h*. The corresponding magnetization curves that are shown below in Fig. 3a exhibit a linear increase with *h* at low fields and have a slope (uniform susceptibility) increasing with temperature. The excitonic condensation is reflected by a kink at *h*_*c*_(*T*) in the *M*(*h*, *T*) curves at constant temperature. The complete *h*-*T* phase diagram in Fig. 2b shows a clear increase of *h*_*c*_ with increasing temperature.

**Figure 1:**
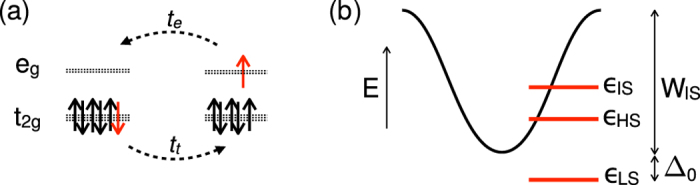
(**a**) Nearest-neighbour hopping process that gives rise to IS-LS exchange. (**b**) Cartoon of the atomic multiplet energies together with the dispersion of a single IS state on the LS background.

**Figure 2:**
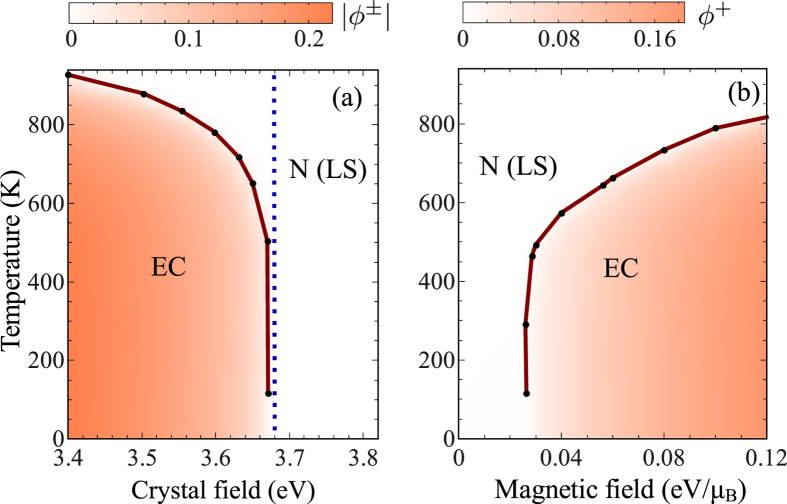
Dependence of the critical temperature *T*_*c*_ on the crystal-field splitting at *h* = 0 (**a**) and on the magnetic field *h* at Δ = 3.68 (**b**). Other parameters are *U* = 4, *J* = 1, *t*_*aa*_ = 0.4118, *t*_*bb*_ = −0.1882, and *t*_*ab*_ = 0.05.

**Figure 3:**
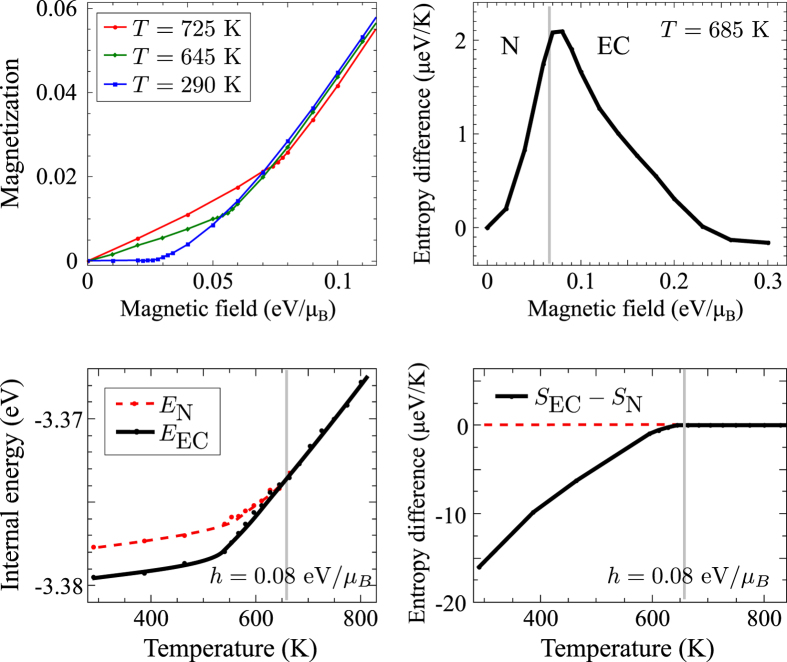
Dependencies of the magnetization and the entropy difference on the strength of the external magnetic field (upper row) and dependencies of the internal energy *E* and the entropy difference on the temperature at constant magnetic field (lower row). Other parameters are taken the same as in Fig. 2.

The behavior of the present model is easily understood by considering the strong coupling limit. At *T* = 0 and *h* = 0 the excitation spectrum is described by the exciton band with a finite gap (see Fig. 1). At nonzero field *h*, the exciton band experiences the Zeeman splitting, thus as soon as the gap closes, the condensate starts to develop. At nonzero *T*, a larger *h*_*c*_ is required to form EC in order to overcome the higher entropy of the normal phase, see Fig. 3. The entropy differences are calculated accordingly to the thermodynamic relations 
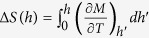
 and 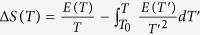. The increase of the uniform susceptibility in the normal phase with temperature reflects the single-ion physics of thermal population of the spinful states. We point out that even in the presence of an external magnetic field the excitonic condensation is a phase transition in the thermodynamic sense as it breaks the spin-rotational symmetry around the direction of the external field (*z*-axis in the present model).

**Figure 4:**
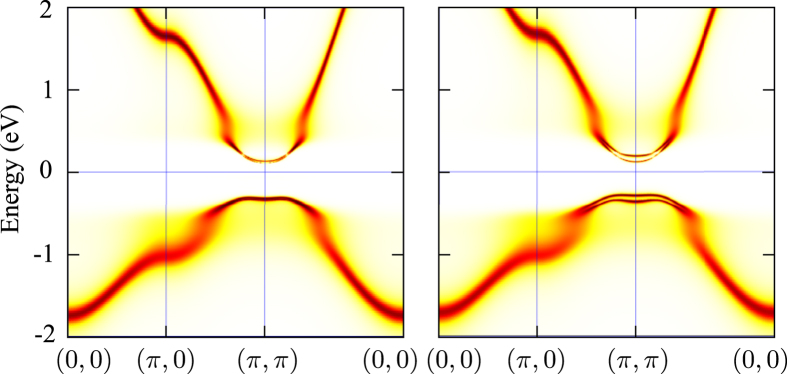
One-particle spectral densities *A*(**k**, *ω*) calculated with the maximum-entropy method^29^ in the normal (left) and EC (right) phases of Fig. 2(b) at *T* = 290 K, *h* = 0.02 eV/*μ*_B_ and *h* = 0.05 eV/*μ*_B_, respectively.

In order to demonstrate that the field-induced transition is not connected to any significant changes or closing of the one-particle gap we have calculated the one-particle spectra above and below *h*_*c*_. The spectra in Fig. 4 show that the Zeeman splitting of the electronic bands is order of magnitude smaller than the charge gap.

**Figure 5:**
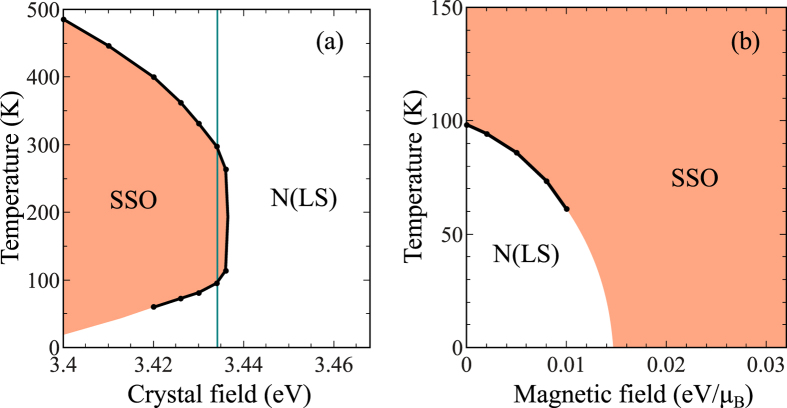
Dependence of the transition temperature between LS homogeneous and HS-LS disproportionated (SSO) phases on the crystal-field splitting at *h* = 0 (**a**) and on the magnetic field *h* at Δ = 3.434 (**b**). Other parameters are *U* = 4, *J* = 1, *t*_*aa*_ = 0.45, *t*_*bb*_ = 0.05, and *t*_*ab*_ = 0.

Next, we discuss the alternative SSO scenario. To this end we chose the asymmetric hopping parameters of ref. 11 and a crystal field Δ close to the “tip of the belly” in Fig. 5a, for which the SSO transition is reentrant and the exciton condensation is suppressed^21^. The strong coupling limit provides a simple understanding of the reentrant behaviour^11^. Starting from the purely LS state at *T* = 0, atoms in the HS state are generated randomly with increasing temperature. At the lower *T*_*c*_ the concentration of HS sites becomes so high that the HS-HS repulsion^21^ drives the system into the ordered state that eventually melts at the upper *T*_*c*_. In an external magnetic field *h*, the solid phase expands. This is particularly pronounced for the low-*T* transition from the normal to the solid phase, for which *T*_*c*_ becomes more suppressed with increasing field *h* as shown in Fig. 5b. The sign *dh*_*c*_/*dT* is related to the fact that the transition from the low-*T* (normal) to the high-*T* (solid) phase is driven by the internal energy but not the entropy.

The temperature scales of the SSO and EC transitions are controlled by the values of parameters 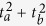 and *t*_*a*_*t*_*b*_, respectively^16^. Varying the ratio *t*_*a*_/*t*_*b*_ one can therefore manipulate the extent of the SSO and EC phases^21^,^22^. While the present model captures the essential physics of the field-induced transition, it is too simplified to provide quantitative estimates of transition temperatures for the real material. In particular, the orbital degeneracy in the real material and non-local fluctuations, which are absent in DMFT treatment, reduce the EC transition temperature.

## Material

The main difference of real LaCoO_3_ from the studied model is the orbital degeneracy, which gives rise to excited states with both *S* = 1 (IS) and *S* = 2 (HS). At low temperatures, when the concentration of excitations is low, the key difference between the IS and HS excitations is their mobility on the LS background. First, we estimate the amplitude for propagation of the IS excitations given by the second-order hopping process shown in Fig. 1. We consider IS excitations with the *T*_1*g*_ orbital symmetry, which have lower excitation energy and higher mobility than their *T*_2*g*_ counterparts^23^,^24^. The *T*_1*g*_ excitation can be viewed as a bound pair of the butterfly-shaped *t*_2*g*_ hole and *e*_*g*_ electron rotated by 45°, i.e., *xy* ⊗ *x*^2^ − *y*^2^, *yz* ⊗ *y*^2^ − *z*^2^ and *zx* ⊗ *z*^2^ − *x*^2^. This geometry makes the *T*_1*g*_ excitations mobile in the plane of the orbitals with practically no hopping in the perpendicular direction. The in-plane nearest neighbour hopping amplitude can be estimated as


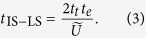


Here, *t*_*t*_ = −0.45 eV and *t*_*e*_ = −0.16 eV are the electronic *xy* ↔ *xy* and *x*^2^ − *y*^2^ ↔ *x*^2^ − *y*^2^ hopping amplitudes, respectively, in the *x* or *y* directions. The numerical values were obtained from *d*-only Wannier functions calculated in the idealized cubic structure^25^,^26^,^27^. The parameter 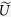 is approximately *U* − 2*J* and we estimate its value for *d*-only model to be in the range of 2–4 eV. This leaves us with *t*_IS−LS_ in the range of 36–72 meV and the bandwidth of IS dispersion indicated in Fig. 1b (considering four in-plane nearest neighbours) in the range of 290–580 meV. Absence of the condensed state for *h* = 0 implies a finite excitation gap and puts the energy of atomic IS excitation to at least half the bandwidth, i.e., 4*t*_IS−LS_. This estimate is consistent with the experimental measurements^28^ and allows to place the energy of atomic HS excitation 100–150 meV below the energy of atomic IS excitation but still above the bottom of the estimated IS excitonic dispersion.

Finally, we analyse the possibility of condensation of the HS bi-excitons. To this end, we view the HS state as a bound pair of two *T*_1*g*_ IS states with different orbital characters. This leads to each of the three HS orbital states to have a sizeable hopping only along one of the cubic axes with the amplitude roughly estimated by


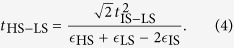


The upper bound for 
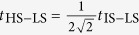
 is obtained assuming 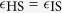 and 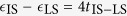 (dictated by the stability of the normal state in the absence of the external field). This yields the bandwidth of the HS dispersion a factor of 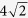 smaller than that of the IS dispersion. A more realistic assumption 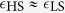 yields an order of magnitude difference between the IS and HS bandwidths. Should the field-induced transition involve HS bi-excitons, their low mobility makes EC an unlikely competitor with SSO driven by HS-HS repulsion of the order of 70 meV^8^.

Therefore, the present results suggest the picture of LaCoO_3_ as a gas of mobile IS excitons on the background of LS ground states interacting via attractive interaction, which leads to formation of immobile bi-excitations, the HS states. The high mobility of the IS excitons allows the lowest excitation of the system to be a wave-like IS state despite the likely 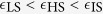 order of the atomic excitation energies. Wave-like character of the low-energy excitations may explain the absence of spin-state (and Co-O bond-length) disproportionation in the intermediate temperature range. The fact that these excitations do not carry charge is consistent with the insulating behaviour in this regime. As the thermally induced concentration of IS excitations grows, bi-excitations, HS states, are formed. The on-set of the bad metallic state shall be viewed of melting of the excitons into free electrons and holes. Quantitative investigation of the present scenario is beyond the scope of this paper. We are not aware of any studies that take the high mobility of the IS excitations into account. In particular, DMFT calculations^8^,^9^ do not include the IS mobility in the normal state, since this involves two-fermion inter-site correlations. Nevertheless, DMFT can capture the field-induced exciton condensation when the correlations become static.

## Conclusions

We have used two-orbital Hubbard model to simulate the effect of an external magnetic field on the ordering transition in the vicinity of spin-state crossover. We find that the *dh*_*c*_/*dT* > 0 slope observed in LaCoO_3_ is consistent with exciton condensation, but inconsistent with SSO. We show that the field-induced transition is of the insulator-to-insulator type. We have estimated dispersion of the IS and HS excitations of the LS ground state and found sizeable bandwidths for the IS excitations of the order of several 100 meV, while the bandwidth of the HS excitations is an order of magnitude smaller. We conclude that the field-induced transition is a Bose-Einstein condensation of the IS excitons. The mobility of IS excitations is a key property of the low-temperature regime that has to be taken into account in description of LaCoO_3_.

## Additional Information

**How to cite this article**: Sotnikov, A. and Kuneš, J. Field-induced exciton condensation in LaCoO_3_. *Sci. Rep.* **6**, 30510; doi: 10.1038/srep30510 (2016).

## References

[b1] Zobel C. *et al.* Evidence for a low-spin to intermediate-spin state transition in LaCoO_3_. Phys. Rev. B 66, 020402 (2002).

[b2] Knzíek K., Jirák Z., Hejtmánek J., Novák P. & Ku W. GGA + *U* calculations of correlated spin excitations in LaCoO_3_. Phys. Rev. B 79, 014430 (2009).

[b3] Yamaguchi S., Okimoto Y., Taniguchi H. & Tokura Y. Spin-state transition and high-spin polarons in LaCoO_3_. Phys. Rev. B 53, R2926 (1996). 10.1103/physrevb.53.r2926 9983886

[b4] Abbate M., Potze R., Sawatzky G. A. & Fujimori A. Band-structure and cluster-model calculations of LaCoO_3_ in the low-spin phase. Phys. Rev. B 49, 7210 (1994). 10.1103/physrevb.49.7210 10009458

[b5] Korotin M. A. *et al.* Intermediate-spin state and properties of LaCoO_3_. Phys. Rev. B 54, 5309 (1996). 10.1103/physrevb.54.5309 9986488

[b6] Knížek K., Novák P. & Jirák Z. Spin state of LaCoO_3_: Dependence on CoO_6_ octahedra geometry. Phys. Rev. B 71, 054420 (2005).

[b7] Eder R. Spin-state transition in LaCoO_3_ by variational cluster approximation. Phys. Rev. B 81, 035101 (2010).

[b8] Zhang G., Gorelov E., Koch E. & Pavarini E. Importance of exchange anisotropy and superexchange for the spin-state transitions in *R*CoO_3_ (*R* = rare earth) cobaltates. Phys. Rev. B 86, 184413 (2012).

[b9] Křápek V. *et al.* Spin state transition and covalent bonding in LaCoO_3_. Phys. Rev. B 86, 195104 (2012).

[b10] Bari R. A. & Sivardière J. Low-spin-high-spin transitions in transition-metal-ion compounds. Phys. Rev. B 5, 4466 (1972).

[b11] Kuneš J. & Křápek V. Disproportionation and metallization at low-spin to high-spin transition in multiorbital Mott systems. Phys. Rev. Lett. 106, 256401 (2011). 21770658 10.1103/PhysRevLett.106.256401

[b12] Karolak M., Izquierdo M., Molodtsov S. L. & Lichtenstein A. I. Correlation-driven charge and spin fluctuations in LaCoO_3_. Phys. Rev. Lett. 115, 046401 (2015). 26252698 10.1103/PhysRevLett.115.046401

[b13] Altarawneh M. M. *et al.* Cascade of magnetic field induced spin transitions in LaCoO_3_. Phys. Rev. Lett. 109, 037201 (2012). 22861888 10.1103/PhysRevLett.109.037201

[b14] Rotter M. *et al.* Mechanism of spin crossover in LaCoO_3_ resolved by shape magnetostriction in pulsed magnetic fields. Sci. Rep. 4, 7003 (2014). 25384532 10.1038/srep07003 PMC4227009

[b15] Ikeda A. *et al.* Spin state ordering of strongly correlating LaCoO_3_ induced at ultrahigh magnetic fields. Phys. Rev. B 93, 220401 (2016).

[b16] Kuneš J. & Augustinský P. Excitonic instability at the spin-state transition in the two-band Hubbard model. Phys. Rev. B 89, 115134 (2014).

[b17] Georges A., Kotliar G., Krauth W. & Rozenberg M. J. Dynamical mean-field theory of strongly correlated fermion systems and the limit of infinite dimensions. Rev. Mod. Phys. 68, 13 (1996).

[b18] Werner P., Comanac A., de’ Medici L., Troyer M. & Millis A. J. Continuous-time solver for quantum impurity models. Phys. Rev. Lett. 97, 076405 (2006). 17026256 10.1103/PhysRevLett.97.076405

[b19] Gull E. *et al.* Continuous-time Monte Carlo methods for quantum impurity models. Rev. Mod. Phys. 83, 349 (2011).

[b20] Kuneš J. Phase diagram of exciton condensate in doped two-band Hubbard model. Phys. Rev. B 90, 235140 (2014).

[b21] Kuneš J. Excitonic condensation in systems of strongly correlated electrons. J. Phys. Condens. Matter 27, 333201 (2015). 26218828 10.1088/0953-8984/27/33/333201

[b22] Tatsuno T., Mizoguchi E., Nasu J., Naka M. & Ishihara S. Magnetic field effects in a correlated electron system with spin-state degree of freedom - Implication of an excitonic insulator. ArXiv e-prints. Available at: http://arxiv.org/abs/1606.01681 (2016).

[b23] Dresselhaus M. S., Dresselhaus G. & Jorio A. In Group Theory: Application to the Physics of Condensed Matter, Ch. 5, 79–95 (Springer-Verlag Berlin Heidelberg, 2008).

[b24] Kuneš J. & Augustinský P. Excitonic condensation of strongly correlated electrons: The case of Pr_0.5_Ca_0.5_CoO_3_. Phys. Rev. B 90, 235112 (2014).

[b25] Schwarz K. & Blaha P. Solid state calculations using WIEN2k. Comput. Mater. Sci. 28, 259 (2003).

[b26] Kuneš J. *et al.* Wien2wannier: From linearized augmented plane waves to maximally localized Wannier functions. Comput. Phys. Commun. 181, 1888 (2010).

[b27] Mostofi A. A. *et al.* Wannier90: A tool for obtaining maximally-localised Wannier functions Comput. Phys. Commun. 178, 685 (2008).

[b28] Haverkort M. W. *et al.* Spin state transition in LaCoO_3_ studied using soft X-ray absorption spectroscopy and magnetic circular dichroism. Phys. Rev. Lett. 97, 176405 (2006). 17155490 10.1103/PhysRevLett.97.176405

[b29] Jarrell M. & Gubernatis J. Bayesian inference and the analytic continuation of imaginary-time quantum Monte Carlo data. Phys. Rep. 269, 133 (1996).

